# The Stability of TiO_2_ Phases Studied Using r^2^SCAN in the Hubbard-Corrected Density Functional Theory

**DOI:** 10.3390/molecules30030560

**Published:** 2025-01-26

**Authors:** Jared Pohlmann, Manjula Raman, Lily Bonds, Kenneth Park

**Affiliations:** Department of Physics, Baylor University, Waco, TX 76798, USA; jared_pohlmann1@baylor.edu (J.P.); manjula_raman1@baylor.edu (M.R.); bondsli@musc.edu (L.B.)

**Keywords:** density functional theory, Hubbard, meta-GGA, SCAN, TiO2, phase stability

## Abstract

Titanium dioxide is a quintessential transition metal oxide with many technologically important applications. With its richness in phases, it has also been a testing ground for numerous theoretical studies including density functional theory. We investigated several phases of TiO_2_ using the all-electron density functional theory with a regularized–restored strongly constrained appropriately normed (r^2^SCAN) exchange–correlation functional, a popular choice of meta-generalized gradient approximation (meta-GGA). Specifically, the equilibrium lattice parameters were more accurate than those predicted by GGA and agreed well overall with the experimental data. With increasing pressure, the order of stability was determined as anatase < columbite < rutile < baddeleyite < orthorhombic I < cotunnite, as in the calculations using GGA. Including the Hubbard correction term, the correct ordering between rutile, anatase, and columbite can be achieved, consistent with experimental observations. The necessary U value using r^2^SCAN is much smaller than that using GGA+U. In addition, the Hubbard correction method using r^2^SCAN is substantially less sensitive to the size of the local projection space compared to the GGA+U study reported recently. We attribute these significantly improved results to the reduced self-interaction error in the r^2^SCAN functional.

## 1. Introduction

TiO_2_ has attracted a long and sustained interest due to its highly desirable and often intriguing properties with respect to many technologically important applications [1,2,3,4,5,6,7,8,9,10,11,12]. For example, in heterogeneous catalysis, it has been used as a support for metal nanoparticles [4,7,11]. Its synergistic effects on various catalytic reactions, as well as the role of surface defects, have been extensively investigated. The excellent photo-reactivity of TiO_2_ is another subject of intense and ongoing research [6,10]. It has been studied regarding its potential in the photolysis of water [1], degradation of toxic organic molecules [5], gas sensors, and dye-sensitized solar cells [2,8], in part due to its stability, cost effectiveness, and environmental friendliness.

**Figure 1:**
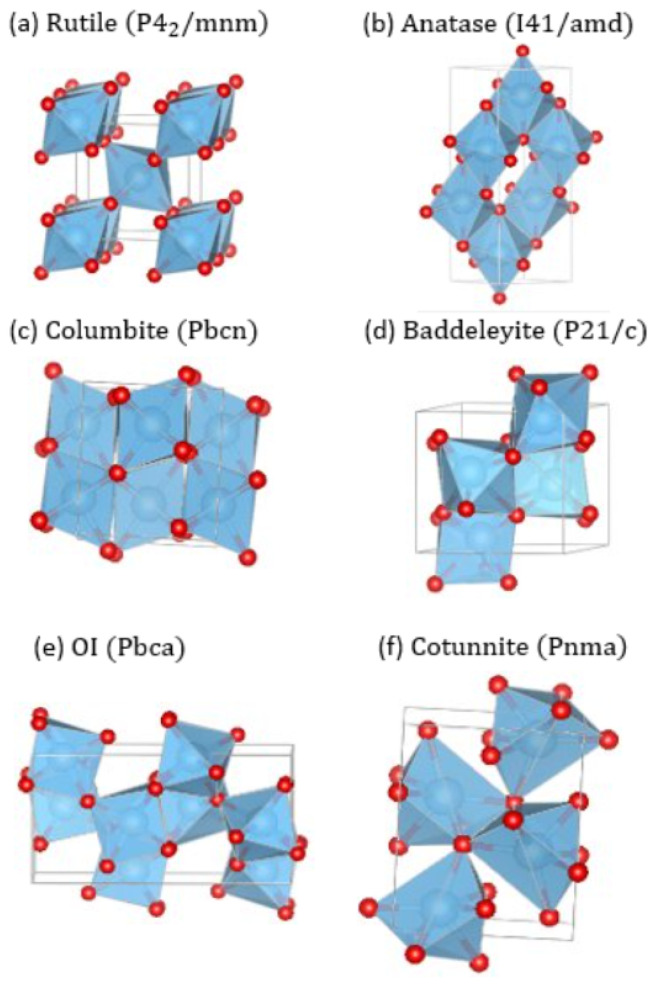
Structures of TiO_2_ phases with Ti (blue) and O (red) are shown using VESTA [26]: (**a**) rutile, (**b**) anatase, (**c**) columbite, (**d**) baddeleyite, (**e**) orthorhombic I, and (**f**) cotunnite.

TiO_2_ also exhibits richness in its phase diagram with various polymorphs. In an ambient condition, rutile $P4_{2}/mnm$ (Figure 1a) and anatase $I4_{1}/amd$ (Figure 1b) are two of the best known stoichiometric and stable forms among several polymorphs [13,14,15,16]. At elevated pressures, they undergo phase transitions to various structures, including α-PbO_2_-type TiO_2_ II, also known as columbite (*Pbcn*) (Figure 1c), monoclinic baddeleyite ($P2_{1}/c$) (Figure 1d), orthorhombic I (*Pbca*) (Figure 1e), and orthorhombic II, also known as cotunnite (*Pnma*) (Figure 1f) [17,18,19]. These high-pressure phases have been examined in comparison to stishovite (SiO_2_) for a better understanding of the Earth’s mantle, partly because TiO_2_ phases allow lower, more easily accessible pressures to induce structural transformation [20]. More recently, it has been reported that the high-pressure phases of several minerals can be stabilized at ambient pressure using a high-pressure torsion method [21,22,23,24,25]. These studies have renewed interest in the possibility of tailoring materials’ properties by synthesizing and quenching high-pressure structures without doping.

The stability of TiO_2_ phases, including high-pressure phases, has been scrutinized using density functional theory (DFT). Using local density approximation (LDA) or generalized gradient approximation (GGA), the general trend of stability and subsequent phase transitions have been successfully reproduced: rutile, anatase, and columbite → baddeleyite → OI → cotunnite, with increasing pressure [27,28,29,30,31,32,33,34,35,36]. However, the standard density functional approximations (DFAs), as well as hybrid functionals, fail to replicate the relative stability among the low-pressure phases, such as rutile, anatase, and columbite. In order to resolve this issue, a number of research groups have explored the Hubbard-corrected DFT approach to achieve the correct ordering of the stability among the low-pressure TiO_2_ phases. For example, Arroyo-de Dompablo et al. [37] reported that the total energy of rutile becomes lower than that of anatase with a U value larger than 5 eV in the GGA+U approach using the Perdew–Burke–Ernzerhof (PBE) functional [38,39]. The authors further suggested that with a range of U values between 5 eV and 8 eV, their study predicted the correct ordering in energetics among the phases, including columbite: $E_{rutile}<E_{anatase}<E_{columbite}$. Similarly, Curnan and Kitchin reported that U values between 4.7 eV and 7.0 eV with the use of the PBE functional reproduced the ordering of the stability among the rutile, anatase, brookite, and columbite phases, consistent with experiments: $E_{rutile}<E_{anatase}<E_{brookite}<E_{columbite}$ [40].

While the DFT+U approach can be attractive due to its computational efficiency, the treatment of the Hubbard U on-site repulsive energy as an empirical parameter is unappealing from a theoretical point of view. More seriously, the sensitivity of the calculated results to the underlying input parameters, such as the size of the local projection space for the correlated *d* orbitals, can be problematic. We recently performed a systematic investigation into the size effect of the local projection space on the PBE+U calculations of rutile and anatase [41]. Our results showed that different ranges of the local projection can produce strikingly different quantitative results for the lattice constants, electronic structures, charge density maps, and the relative stability between rutile and anatase. In particular, if the local projection space for the Hubbard interaction chosen was sufficiently small, even a very large value of U, up to 10 eV, could not reproduce the correct ordering in relative energetics between rutile and anatase, sharply contradicting the aforementioned results. The quantitative discrepancies were attributed to the dependence of the occupancy numbers of the correlated orbitals on the size of the projection space, causing disparate shifts in the orbital-dependent potential energy. Thus, the study highlighted that DFT+U calculations, particularly those using the PBE, should be interpreted with caution.

As another approach to improve upon the standard DFA, meta-GGAs have been developed by including the electronic kinetic energy density in addition to the density *n*(**r**) and its gradient ∇n(**r**). Among them, the more recently reported strongly constrained and appropriately normed (SCAN) functional has been under the spotlight for its ability to accurately describe the structures and energies of diversely bonded systems ranging from molecules to solids [42,43,44,45]. Its success has in part been traced back to the properties of the functional satisfying all known exact constraints for the exchange–correlation term [42]. The use of the kinetic energy density allows SCAN to be more flexible regarding the distinct types of chemical bonding, from metallic to covalent and from hydrogen to van der Waals, while retaining computational efficiency as a semi-local functional. For binary oxides and compounds, SCAN has been demonstrated to be in a better agreement with experimental data than the PBE [46,47,48,49]. Specifically, for the relative stability among TiO_2_ phases, Zhang et al. [47] reported that anatase, brookite, columbite, and β-TiO_2_ were more stable than rutile using SCAN, just as they were when using the PBE. However, the energy differences calculated using SCAN were substantially reduced compared to those calculated using the PBE. The authors further showed that the correct ordering of stability could be achieved with a very modest U value of 2 eV in the SCAN+U approach. They credited the smaller value of U necessary for reproducing the correct energetics to the reduced self-interaction error (SIE) in SCAN [50,51].

In order to better understand the performance of the SCAN functional and its use in the Hubbard-corrected DFT approach, we report DFT calculations of TiO_2_, including high-pressure phases, using a regularized–restored strongly constrained and appropriately normed (r^2^SCAN) functional, a version of SCAN with improved numerical efficiency while maintaining adherence to the exact constraints [52]. With r^2^SCAN implemented self-consistently in all-electron calculations, our goal was to make a systematic comparison with PBE and PBE+U calculations in terms of the equilibrium structures, relative stability, and elastic and electronic properties.

## 2. Results and Discussions

### 2.1. Equilibrium Structures and Phase Stability at 0 GPa

Table 1 lists the optimized structural parameters of TiO_2_ phases calculated using r^2^SCAN and PBE functionals. They are compared with selected experimental and theoretical values reported in the literature. The structures referred to are the equilibrium structures at *p* = 0 GPa unless stated otherwise. Also, the internal coordinates of the optimized structures are provided in Table S1 in the Supplementary Materials. In general, our values calculated using the PBE functional matched closely with previously reported theoretical values for rutile, anatase, and columbite [29,32,35,37,40,53,54,55]. Also, the lattice constants predicted using the r^2^SCAN functional deviated no more than 0.3% from those reported by Zhang et al. using the SCAN functional [53]. The lattice constants calculated using r^2^SCAN (or SCAN) were significantly smaller, typically by up to 1%, than those calculated using the PBE, which is known to overestimate the lattice constants. As a result, the lattice constants of the three low-pressure polymorphs calculated using r^2^SCAN were much closer to the experimental values, as observed in Table 1. For rutile and anatase, the lattice parameters were within 0.2% and 0.9% of the experimentally measured values, respectively. The calculated lattice constants for columbite were also no more than 0.2% away from the experimental values measured under ambient conditions after the columbite phase was pressure-quenched [17,56].

For baddeleyite, the lattice constants calculated using the PBE functional were in excellent agreement with those reported by Fu et al. [32], differing by no more than 0.3%. Similarly, for OI, our lattice constants were practically identical to the compared values when using the same PBE functional (Table 1). However, for cotunnite, the calculated lattice constants varied by a greater extent among the calculations even when using the same GGA level. A difference of about 1.0% for the lattice constants *b* and *c* was observed as compared with the value reported by Fu et al. [32], whereas the values for the lattice constant *a* were in good agreement. On the other hand, our results for cotunnite agreed better with those reported by Niu et al. [54] when using the same PBE functional, with the largest difference of 0.7% for the lattice constant *b*. Our lattice parameters were also compared with the values predicted using another GGA functional (PW91) [29]. In this case, the largest deviation was observed to be about 0.7% as well, but for the lattice constant *a*.

The r^2^SCAN functional reduced the overestimated lattice constants of the high-pressure phases by substantial amounts, just as was observed for the three low-pressure phases. The lattice constants for baddeleyite decreased by 0.5% along *a* and by 1% along *b* and *c*, compared to those calculated using the PBE. The calculated lattice constants also agreed well with the values reported by Zhang et al. calculated using the SCAN functional [53]. Likewise, for OI and cotunnite, the lattice constants decreased by 0.7% to 0.8% and by 0.6% to 0.9%, respectively. For OI and cotunnite, we are not aware of any reported lattice parameters calculated using the SCAN functional.

The structural parameters of the high-pressure phases calculated using the *r*^2^SCAN functional were generally closer to the experimental values than those calculated using the PBE functional as well. For baddeleyite, our calculation using the PBE predicted that the values would deviate from the experimental ones by 4.3%, −1.3%, and 4%, respectively, for *a*, *b*, and *c*. Using *r*^2^SCAN, the deviation decreased to 3.7% for *a* and 3% for *c*, whereas it increased to −2.3% for *b*. Consequently, the equilibrium volume per formula unit, *V*_0_, decreased from 29.98 Å^3^ to 29.23 Å^3^, closer to the experimental value of 28.06 Å^3^ (Table 1). However, it was also noted that the agreements between the theoretical lattice constants, including our own, and the experimental values for baddeleyite were less satisfactory compared to the three low-pressure TiO_2_ phases. We believe that the sizable discrepancy is perhaps in part due to the uncertainty involved in the extrapolation scheme in deducing the zero-pressure lattice constant [20].

For OI, the lattice parameters from the r^2^SCAN calculation at 0 GPa yielded *V*_0_ = 27.80 Å^3^ per formula unit, which was in good agreement with the values ranging from *V*_0_ = 27.27 Å^3^ to *V*_0_ = 27.97 Å^3^ deduced from experiments [18,28,58]. Just like other high-pressure phases, OI is not pressure-quenchable. The experimental lattice constants for OI in Table 1 were based on an X-ray diffraction measurement performed at 28 GPa [18]. In order to simulate the experimental structure, the OI structure was optimized, with the reduced volume corresponding to *p* = 28 GPa (Figure S1 in the Supplementary Materials). The lattice constants of the high-pressure structure calculated using r^2^SCAN were in excellent agreement with the experimental values, differing by no more than 0.2%. These values are clearly better than the ones obtained using the PBE as well as the hybrid B3LYP functionals at 28 GPa [33]. Similarly, for cotunnite, the quoted experimental lattice constants are the ones acquired at 61 GPa [19]. At 0 GPa, the equilibrium volume was estimated to be 15.82 cm^−3^/mol, which is about 23.02 Å^3^ per formula unit [58]. The lattice parameters calculated using r^2^SCAN yielded a better agreement with *V*_0_ = 22.14 Å^3^, in contrast to *V*_0_ = 26.29 Å^3^ calculated using the PBE as well as *V*_0_ = 25.80 Å^3^ calculated by Fu et al. [32] and *V*_0_ = 26.15 Å^3^ calculated by Ma et al. [29] using the same functional.

**Figure 2:**
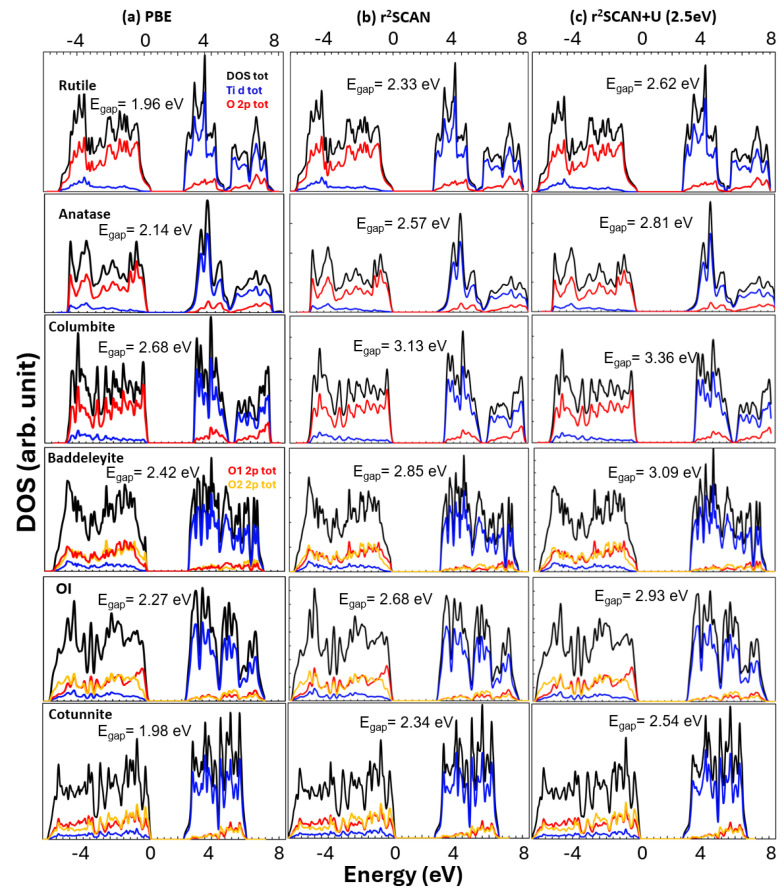
Electronic density of states calculated with $R_{MT}$ (Ti) = 1.78 *a_B_* using (**a**) the PBE, (**b**) r^2^SCAN, and (**c**) r^2^SCAN+U (2.5 eV) functionals for rutile, anatase, columbite, baddeleyite, OI, and cotunnite. The Ti *d* and O *p* states are shown with blue and red curves, respectively, as well as the inequivalent oxygen (if applicable) with yellow. Also, the Kohn–Sham band gap energies are indicated.

The electronic densities of states (DOSs) for the six phases in their equilibrium structures were also investigated using the r^2^SCAN functional. The DOS curves of all TiO_2_ phases show that the occupied states in the valence bands were mostly dominated by O *p* orbitals and the empty conduction bands by Ti *d* orbitals (Figure 2). While TiO_2_ can be characterized as a charge transfer insulator, it has substantial covalency, as evidenced by the overlaps between O *p* and Ti *d* orbitals [59]. For rutile, anatase, columbite, and baddeleyite, the Kohn–Sham band gaps (E_*gap*_) were 2.33, 2.57, 3.13, and 2.85 eV, respectively (Figure 2b), in good agreement with the values reported by Zhang et al. [53]. These values were roughly 0.4 eV greater than those predicted using the PBE functional (Figure 2a). However, they are well short of the experimental values, for example, 3.0 [60] and 3.2 eV [61] for rutile and anatase, respectively, indicating that the r^2^SCAN functional still suffers from the SIE, as in the PBE functional, albeit to a smaller extent [53]. For OI and cotunnite, we are not aware of any reports of the band gap or calculations of the electronic structure using the SCAN functional. Our values of 2.68 and 2.34 eV, respectively, for OI and cotunnite were larger than the 2.27 and 1.98 eV calculated using the PBE functional by about 0.4 eV, as well. The band gap values from the PBE calculations are consistent with an E_*gap*_ estimated to be greater than 2 eV for OI [30] and, for cotunnite, an E_*gap*_ calculated to be 2.02 eV [62].

**Figure 3:**
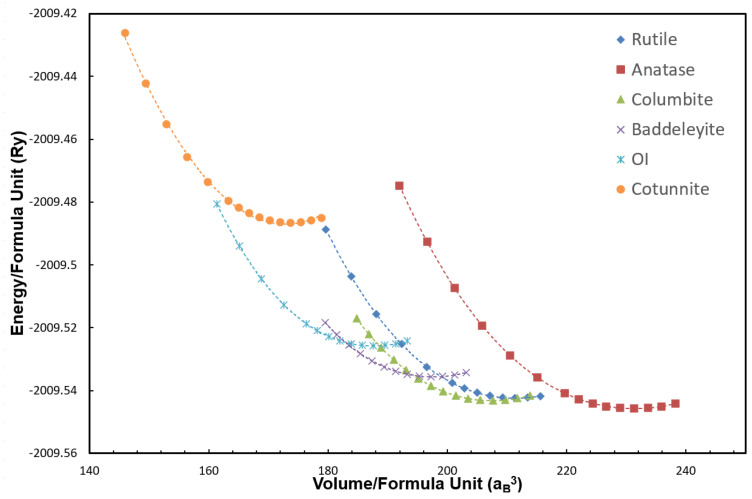
Total energy of TiO_2_ as a function of the volume for various phases calculated using the r^2^SCAN functional. Each symbol represents a full structural optimization: rutile (dark blue diamond), anatase (red square), columbite (green triangle), baddeleyite (purple cross), OI (blue cross), and cotunnite (orange circle). The solid line is fitted with the Birch-Murnaghan equation of state.

Figure 3 presents the total energy of each phase as a function of the volume of the unit cell using the r^2^SCAN functional. For comparison, the energy of each phase was normalized to the energy per TiO_2_ formula unit. At *p* = −(∂*E*/∂*V*) = 0 GPa, rutile, anatase, and columbite lay very close together in terms of their energy, while the other three high-pressure phases were clearly at higher energies. A closer inspection revealed that the energy of anatase was the lowest, followed by that of columbite and then rutile. The relatively small energy differences predicted among the three low-pressure phases can be understood in terms of the structural resemblance that they share [27]. Rutile, anatase, and columbite can all be approximately described as oxygen octahedra with Ti at their centers (Figure 1). They are distinguished by different octahedral linkages: two edge-sharing octahedra along c in rutile, two edge-sharing octahedra forming zigzag chains in columbite, and four edge-sharing octahedra forming zigzag chains in the a–b plane of anatase. The close structural similarity with minute differences in the energetics implies that the phase transformation among them can be quite sensitive to and easily influenced by other factors such as the temperature, residual stress, or history of sample preparations.

The three low-pressure phases were followed by baddeleyite, OI, and cotunnite in the increasing order of the energy at equilibrium. The high-pressure phases exhibit polyhedral shapes that are more severely distorted, with an increased coordination number for Ti (Figure 1). Their energy differences were markedly greater compared to those of the three low-pressure phases. The overall ordering of the energetics was determined as $E_{anatase}<$ $E_{columbite}<$ $E_{rutile}<$ $E_{baddeleyite}<$ $E_{OI}<$ $E_{cotunnite}$. The predicted ordering of the energetics was congruent with what Zhang et al. [53] reported using SCAN: $E_{anatase}<$ $E_{brookite}<$ $E_{columbite}<$ $E_{\beta -TiO_{2}}<$ $E_{rutile}<$ $E_{baddeleyite}$. Between the r^2^SCAN and PBE functionals, the relative energetics of the six phases remained essentially the same, apart from small quantitative differences (Figure S2 in the Supplementary Materials). Our results calculated using the PBE were also in agreement with those reported previously [29,30,32,34,35,62,63].

The dependence of the total energy on the volume further allowed us to explore the system under the condition of hydrostatic pressure. The calculated values were fitted and analyzed with the third-order Birch–Murnaghan equation of state (EOS) [64,65]:

(1) $$p\left(V\right)=-\frac{\partial E}{\partial V}=\frac{3}{2}B_{0}\left[{\left(\frac{V_{0}}{V}\right)}^{7/3}-{\left(\frac{V_{0}}{V}\right)}^{5/3}\right]\{1+\frac{3}{4}\left(B_{0}^{\prime }-4\right)\left[{\left(\frac{V_{0}}{V}\right)}^{2/3}-1\right]\},$$

where *B*_0_ and $B_{0}^{\prime }$ are the bulk modulus and its pressure derivative, respectively, and *V*_0_ is the equilibrium volume. The bulk modulus values calculated using the r^2^SCAN functional were systematically larger than those calculated using the PBE, as one would expect from the smaller lattice constants predicted by r^2^SCAN in contrast to the overestimated lattice constants (Table 1). Also, our bulk modulus values calculated using the PBE were in good agreement with the previously reported values using the same or a similar GGA for the exchange–correlation functional. In comparison with experimental values of the bulk modulus, it is noteworthy that a substantial range of the experimental values could be found for a given phase. This is in part due to the fact that different fitting methods of *V*_0_, *B*_0_, and $B_{0}^{\prime }$ can introduce a substantial amount of uncertainty. This uncertainty can influence the results to the same effect as inherent experimental uncertainty due to different synthesis conditions, hydrostaticity, and the pressure calibration. In particular, Nishio-Hamane et al. [58] pointed out that a very large value of the bulk modulus for cotunnite, e.g., 419 to 485 GPa, can be obtained if a very small value of $B_{0}^{\prime }$, such as 1 to 2, is used. On the other hand, with $B_{0}^{\prime }$ = 4.25, a bulk modulus of 294 GPa is produced. With $B_{0}^{\prime }$ fixed at 4, the Birch–Murnaghan EOS (Equation (1)) becomes the second order, resulting in a bulk modulus of 306 GPa. Given the variability of such fitting procedures, the bulk modulus values obtained using both the r^2^SCAN and the PBE functionals were considered to be in agreement with the reported experimental values.

**Figure 4:**
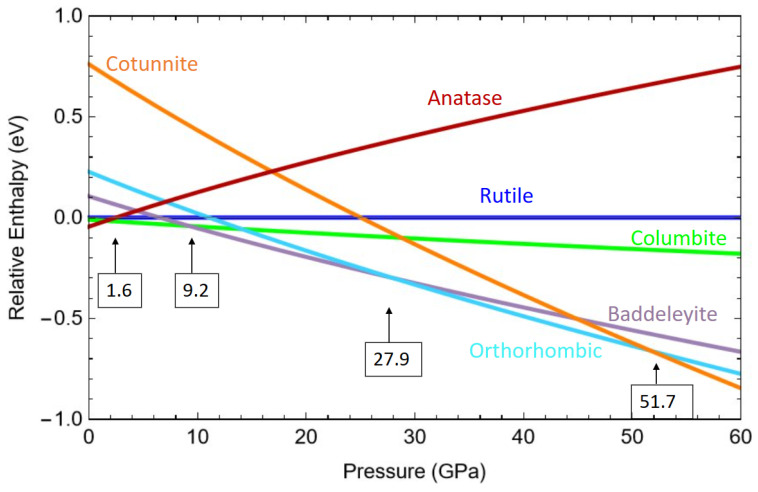
Enthalpy, *H*, of TiO_2_ phases as a function of the pressure calculated from the total energy and volume data using the Birch–Murnaghan equation of state: rutile (dark blue), anatase (red), columbite (green), baddeleyite (purple), OI (blue), and cotunnite (orange). The pressures for the phase transitions are noted in the boxes.

The enthalpy H=E+PV was further calculated to examine the relative stability and phase transitions at an increasing pressure. In Figure 4, the enthalpy of each TiO_2_ phase relative to that of rutile is displayed under hydrostatic pressure from 0 to 60 GPa. At 0 GPa, anatase had the lowest enthalpy among the phases studied in this work. Above 1.6 GPa and until 9.2 GPa, columbite became the most stable phase. This pressure range is in accordance with the observations that anatase transforms into columbite at the low-end limit of the pressure range from 2.3 [66] to 4.5 GPa [17]. It is also consistent with experimental reports in which columbite is typically formed from baddeleyite at around 7 or 8 GPa upon decompression [17,20,28]. From about 9.2 GPa to 27.9 GPa, baddeleyite had the lowest enthalpy. The large pressure range and the transition pressures agreed well with the transformations involving baddeleyite reported by various groups. It has been observed that baddeleyite transforms into columbite at 8 GPa upon decompression [17,20,28]. On the other hand, baddeleyite can convert to OI at as low as 25 GPa upon compression, as reported by Al-Khatatbeh et al. [28]. Between 27.9 and 51.7 GPa, the enthalpy calculated using r^2^SCAN predicted OI to be the most stable phase, and it predicted cotunnite to be the most stable for pressure beyond 51.7 GPa. The transition pressure between OI and cotunnite was in good agreement with the observed transformation of the OI phase into cotunnite taking place between 49.0 and 56.0 GPa [28]. Thus, for the six phases studied in this work, the sequence of the phase transitions was calculated to be anatase ⟶ columbite ⟶ baddeleyite ⟶ OI ⟶ cotunnite, with rutile being slightly less stable than anatase at ambient pressure.

### 2.2. The Effect of the Hubbard Correction

The addition of the Hubbard correction term to the r^2^SCAN functional results in an increase in the band gap as the most notable change. Figure 2c shows that the band gap energies of all TiO_2_ phases increased by approximately 0.2 to 0.3 eV upon U = 2.5 eV being applied to the r^2^SCAN functional calculations. The band gaps for rutile, anatase, columbite, and baddeleyite were now 2.62, 2.81, 3.36, and 3.09 eV, respectively. The increase in the band gap energy was in accordance with a similar increase reported based on SCAN+U(2eV) calculations [53]. The increase in or opening up of a band gap is understood to be a direct consequence of on-site electron–electron repulsion favoring electron localization as it prevails over the delocalization caused by the kinetic energy term. Although the increased band gap energies were still smaller than the experimental values, they represent improvements as one of the original objectives envisioned in the DFT+U approach [67].

**Figure 5:**
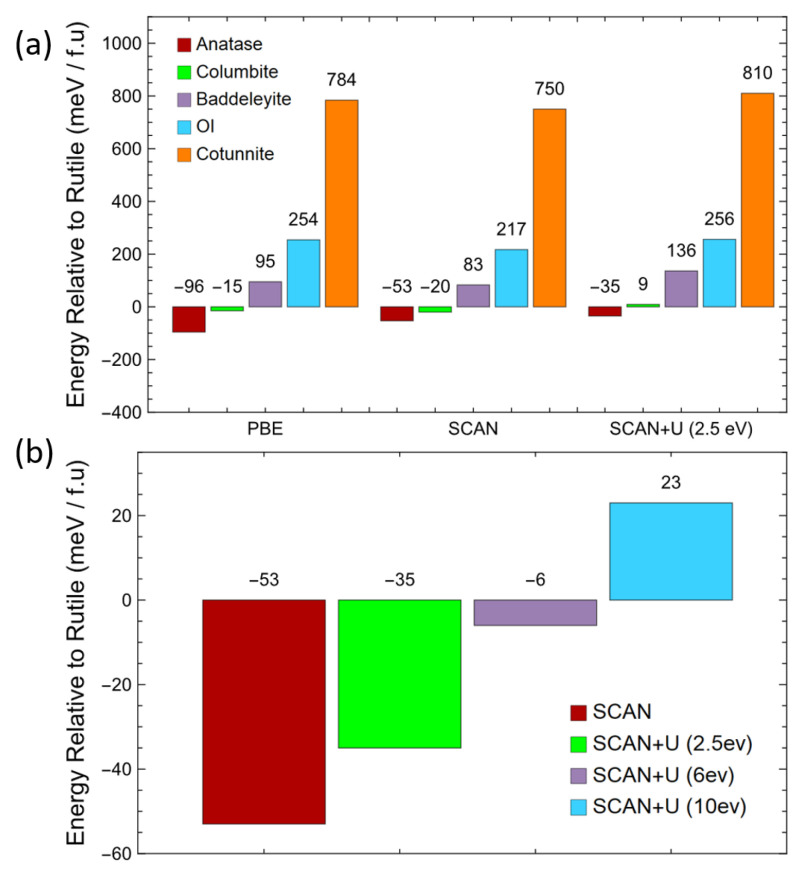
(**a**) The energy of TiO_2_ phases relative to that of rutile (meV per formula unit) calculated with the PBE, r^2^SCAN exchange–correlation functionals, and r^2^SCAN+U (2.5 eV). (**b**) The energy of anatase relative to that of rutile with increasing U values: 2.5, 6, and 10 eV in r^2^SCAN+U.

The relative energetics among the TiO_2_ phases can be considerably influenced by the Hubbard correction as well. In Figure 5a, the total energies of the six phases calculated with r^2^SCAN with the Hubbard U correction are quantitatively compared to those calculated with r^2^SCAN and the PBE at 0 GPa. Using r^2^SCAN, anatase and columbite were slightly lower in energy compared to rutile by −53 and −20 meV, respectively. These values were in a similar range to the previously reported values of −25 and −11 meV [53]. Although r^2^SCAN incorrectly predicted the relative stability among the three low-pressure phases, it is noticeable that the stability gap between rutile and anatase was reduced to −53 meV from the −96 predicted using the PBE. The inclusion of the Coulomb repulsion U = 2.5 eV for *d* orbitals raised the energy for anatase and columbite by 18 and 29 meV, respectively, relative to that of rutile. The increase in energy for columbite was apparently sufficient to make it unstable with respect to rutile. However, the energy of anatase still remained lower than that of rutile. For baddeleyite, OI, and cotunnite, the Hubbard correction increased their total energies by about 40 to 60 meV relative to the energy of rutile. Since they were well separated in terms of their relative energetics as well as in terms of the significantly higher energy in the low-pressure phases, the effect of the Hubbard U was to push up their total energies more or less uniformly.

The relative stability of rutile and anatase could be further altered with an increasing value of U in the r^2^SCAN+U calculations. With a U value increased to 6 eV (Figure 5b), anatase was still more stable than rutile but by merely 6 meV. After U was increased to 10 eV, rutile finally became more stable than anatase by 23 meV. The apparently larger U value, which was necessary to reverse the relative stability between rutile and anatase, is in clear contrast to the much smaller U = 2 eV needed to produce the correct ordering reported by Zhang et al. [53].

In our recent study of TiO_2_ using the PBE+U approach, we reported that the size of the local projection in the application of the Coulomb interaction can play a crucial role in quantitative assessments [41]. After comparing several studies of rutile and anatase calculated using the same PBE+U approach with various DFT codes, we concluded that systematic discrepancies in the structural parameters, electronic structures, charge density distribution, and energetics can be traced to the different effective ranges of the local projection space used to apply the Hubbard correction. This sensitivity directly results from the formulation as the local orbital occupancy *n* (Equations (5) and (6) in Section 3) depends on the size of the local projection space. In an APW method, such as the one used in this study, the local projection space is defined by the augmented sphere. Moreover, it can be varied independently using the muffin-tin radius $R_{MT}$. In the pseudopotential approach using the projector-augmented wave (PAW) method, the local density matrix is usually calculated using the pseudo-wavefunction and the projection function within the PAW radius. However, the PAW radius is not generally an independent parameter because it varies with different pseudopotentials, which in turn depend on the valence electron configuration as well as the inclusion or exclusion of semicore electrons, among other factors. In the SCAN+U study, Zhang et al. [53] used the PAW potential, with Ti 3s and 3p treated as valence states. The corresponding $R_{PAW}$ s 2.3 *aB*, which is much larger than the $R_{MT}$ = 1.78 *aB* used in this study.

**Figure 6:**
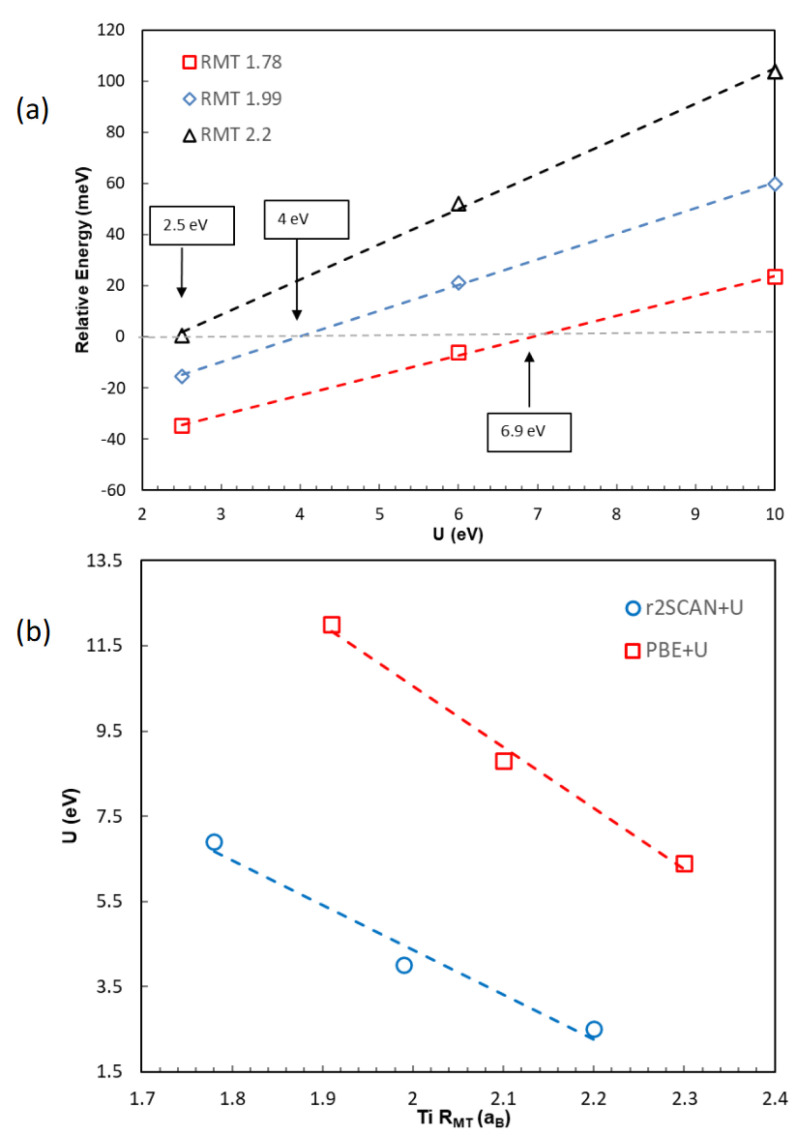
(**a**) The energy of anatase relative to rutile (meV per formula unit) calculated using the r^2^SCAN+U method with different $R_{mt}$ (Ti) values: 1.78 (red square), 1.99 (blue diamond), and 2.2 *a_B_* (black triangle). The estimated minimum U values resulting in a greater stability for rutile are indicated. (**b**) The minimum U values for the correct relative energetics between rutile and anatase were compared using the r^2^SCAN+U (blue circle) and the PBE+U (red square) methods. The dashed lines are only there to guide the eyes.

In order to investigate the effect of the local projection size on the relative stability calculated using the SCAN+U approach, the total energies of rutile and anatase were compared using different muffin-tin radii for Ti as well as different U values. Figure 6 shows the total energy of anatase relative to that of rutile as U was increased in three different sets of $R_{mt}$ (Ti) values: 1.78, 1.99, and 2.2 *aB*. With the smallest value of $R_{mt}$ (Ti) = 1.78 *aB*, it was estimated that anatase would become less stable than rutile after U was increased to 6.9 eV (Figure 6a red). As the $R_{mt}$ (Ti) value was increased to 1.99 *aB*, anatase became less stable at a substantially smaller U value of about 4 eV (Figure 6a, blue). With the largest $R_{mt}$ (Ti) of 2.2 *aB* tested in this study, the energy of anatase was raised above that of rutile at U = 2.5 eV or higher (Figure 6a, black). The U value calculated with the large projection space was comparable to the value reported of U = 2 eV [53]. The results unambiguously establish the dependence of the relative energetics on the size of the Hubbard projection space, calculated using r^2^SCAN+U just as was previously shown using PBE+U [41].

While the Hubbard-corrected SCAN calculations similarly suffered from a dependence on the size of the projection space, there was a clear distinction between the present results and the previous ones calculated using PBE+U. Using the same size of the projection space ($R_{mt}$ (Ti) = 1.78 *aB*), the PBE+U calculations predicted that anatase would remain more stable with respect to rutile even after U was increased to 10 eV. The minimum U value that would reverse the relative stability between rutile and anatase was estimated to be well beyond 10 eV, much larger than the 6.9 eV predicted using r^2^SCAN (Figure 6a). In order to compare the minimum U values between the r^2^SCAN+U and PBE+U calculations necessary to produce the correct relative stability, the $R_{mt}$ (Ti) values of 1.91, 2.1, and 2.3 *aB* were chosen for the PBE+U calculations (Figure 6b). With $R_{mt}$ (Ti) = 1.91 *aB*, the minimum U value was estimated at 12 eV. As the $R_{mt}$ (Ti) was further increased to =2.1 and 2.3 *aB*, the minimum U value for the correct ordering of energetics between rutile and anatase decreased to 8.8 and 6.4 eV, accordingly. These values were consistently larger than what the r^2^SCAN+U calculations predicted, by about 5 to 7 eV, at comparable sizes for the local projection space. To understand why a relatively small value of U was sufficient for the r^2^SCAN+U calculations, the Bader charges were first examined and compared between the calculations using the r^2^SCAN and the PBE functionals [68,69].

Table 2 shows that the Bader charges for Ti and O were +2.28*e* and −1.14*e* for rutile and +2.26*e* and −1.13*e* for anatase, respectively, when calculated using the PBE functional. A bond critical point (BCP) (3, −1) [68] was found approximately midway from Ti to apical O along the Ti-O(1) bond as well as from Ti to equatorial O along the Ti-O(2) bond. With r^2^SCAN, the Bader charges for Ti and O were predicted to be significantly larger in magnitude, e.g., +2.38*e* and −1.19*e* for rutile and +2.37*e* and −1.19*e* for anatase, respectively. The BCP values along the Ti-O bond directions were still found near the midpoints but were slightly smaller, reflecting the shortened bond lengths. Larger Bader charges from the r^2^SCAN calculations indicated a significantly greater charge transfer from Ti to O, leaving much fewer residual electrons at the cation. Fewer electrons remaining at Ti were further confirmed by the local projection of partial charges within the muffin-tin sphere. The charge analysis showed that the *d* occupancies of all TiO_2_ phases calculated using the r^2^SCAN functional were indeed smaller than those calculated using the PBE functional (Supplementary Materials, Table S2). Thus, the r^2^SCAN functional yielded more ionic characteristics for both the rutile and anatase phases of TiO_2_ compared to the PBE functional, even without the Hubbard correction applied.

**Figure 7:**
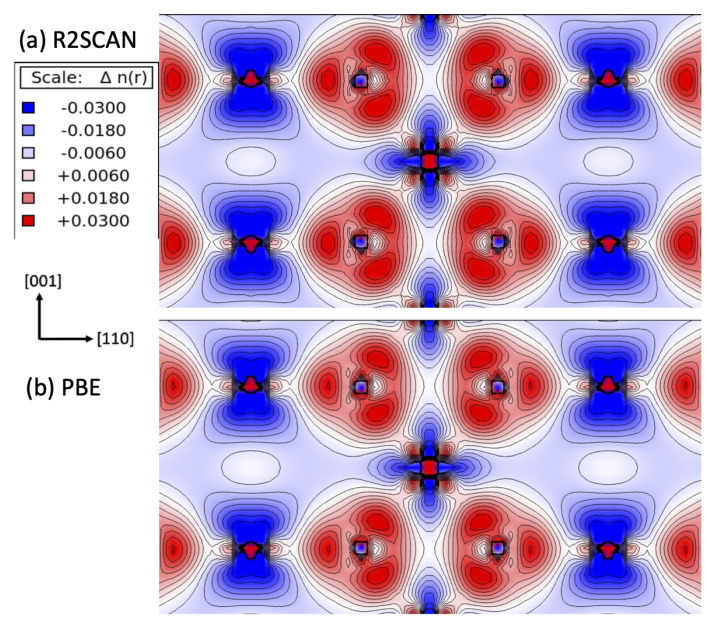
The difference electron density plots for rutile in the (110) plane using (**a**) r^2^SCAN and (**b**) the PBE functional. Blue indicates an electronic charge deficiency and red indicates a surplus.

The stronger ionic nature predicted from the r^2^SCAN calculations can be also appreciated by studying the difference electron density plot, which displays the difference in the electron density between the crystal and the superposed atoms. In Figure 7, the difference electron density plots for rutile are compared using (a) the r^2^SCAN and (b) the PBE functionals. With the r^2^SCAN functional, the $sp^{2}$-type bonding regions centered at O in the planar O-Ti_3_ cluster were clearly enlarged, indicating a greater electron excess around O. Correspondingly, the charge-deficient regions (blue) at Ti, reflecting the $t_{2g}$-type orbital symmetry ($d_{xy}$, $d_{yz}$, $d_{zx}$), were likewise enhanced. In addition, the excess charge densities were diminished along the Ti-O bond directions: Ti-O(1) (apical O) and Ti-O(2) (equatorial O). The analysis of the Ti partial charges, calculated by integrating the projected density of states (PDOS), indeed revealed that the occupation numbers in the *eg*-type orbital symmetry ($d_{z^{2}}$, $d_{x^{2}-y^{2}}$), as well as in the total *d* population, were considerably lower than what the PBE predicted (see Table S3 and Figure S3 for rutile and Table S4 and Figure S4 for anatase in the Supplementary Materials).

The redistribution of charges around Ti and O observed in the r^2^SCAN calculations can be understood as a consequence of the improved exchange–correlation energy using the dimensionless kinetic energy variable $\bar{\alpha }$. The variable $\bar{\alpha }$ (c.f. α in SCAN) measures the deviation in the charge distribution from that of a single orbital (Equation (2) in Section 3) [52]. Thus, it is closely related to the electron density and the electron localization function [70]. In the atomic shell regions of a high electron density, α is much less than 1, as demonstrated in closed-shell atomic systems, e.g., Ar_2_ [71] and Kr_2_ [52], as well as hydrogenic anions [72]. In the $sp^{2}$-type bonding region centered at the anionic O^2−^ of TiO_2_ (Figure 7), α is expected to be less than 1 as well, with a small density gradient, *s*, and a high electron density. This is the regime in which the SCAN exchange enhancement factor $F_{x}\left(s,\alpha \right)$ is enhanced compared to the one used in the PBE with α<1 and a small *s* [42]. Since the exchange energy $E_{x}\left[n\right]=\int d^{3}rn\varepsilon_{x}^{unif}\left(n\right)F_{x}\left(s\right)$ is made negative by a construction with $\varepsilon_{x}^{unif}\left(n\right)=-\pi {\left(3\pi^{2}n\right)}^{1/3}<0$ and $F_{x}\left(s\right)>0$, the total energy is lowered, with a larger charge accumulation at O and hence more depletion at Ti.

Similarly, the charge reduction along the Ti-O bond directions observed in the r^2^SCAN calculation (Figure 7a) can be explained by the changes in the enhancement factor caused by α. The bond center between Ti and O is analogous to a region of density overlap between closed shells. With a relatively small density gradient, the von Weizsäcker kinetic energy density should be small, e.g., $\tau^{W}\approx 0$. Consequently, α becomes like $\tau /\tau^{unif}\propto n/n^{5/3}$, which results in α≫1 with n→0 [71]. In this case, the exchange enhancement factor in r^2^SCAN is expected to be smaller than that used in the PBE [42].

Along with the redistribution of charges, the improvement in predicting the lattice parameters over the PBE can be traced back to the modified enhancement factor for the r^2^SCAN exchange correlational functional as well. The typical overestimation of the bond lengths in various GGAs has been understood as a consequence of density inhomogeniety (e.g., a larger density gradient, *s*) favored by the gradient corrections [73,74]. The enhancement factor $F_{x}$ in r^2^SCAN does not increase monotonically, unlike in GGA [42]. Rather it remains at the much smaller conjectured value of 1.174 for all values of α, in contrast to the general Lieb–Oxford bound of 1.804 used in the PBE [75]. These changes in the enhancement factor, along with the flexibility afforded by α, enable the SCAN calculations to alleviate the systematic overestimation of the lattice constants observed in the PBE.

Table 3 compares various bond lengths calculated using the r^2^SCAN and the PBE functionals for rutile and anatase. The bond lengths of 1.986 Å and 1.987 Å between Ti and apical oxygen O(1) for rutile and anatase, respectively, were significantly shorter than 2.005 Å and 2.004 Å. Likewise, the bond lengths of 1.947 Å and 1.934 Å between Ti and equatorial oxygen O(2) for rutile and anatase, respectively, were also closer to the experimental values. Furthermore, it should be noted that the distances between planar oxygen anions were decreased, for example, in rutile from 2.560 Å calculated using the PBE to 2.535 Å calculated using r^2^SCAN, very close to the experimental value of 2.537 Å. The shortened oxygen anion distance is consistent with the capability of SCAN to describe properly non-covalent bonds between two closed shells, for example, van der Waals interactions among highly polarizable oxygen anions [71].

The Hubbard-corrected DFT approach based upon r^2^SCAN also reaps the benefits of the improvement that the exchange–correlation functional brings. A more ionic nature for TiO_2_ calculated using the r^2^SCAN functional allows for a much smaller Hubbard U value in order to achieve a comparable effect to that observed in the PBE+U method. The Hubbard correction raises the *d* orbital energies according to their occupancies (Equation (6) in Section 3). For a band less than half-filled, such as in TiO_2_, the correction empties the localized band, subsequently increasing the ionicity. With a very modest value of U = 2.5 eV, the Bader charges for Ti and O increased to +2.46*e* and −1.23*e*, respectively, for rutile and similarly to +2.44*e* and −1.22*e*, respectively, for anatase in the r^2^SCAN+U calculations (Table 2). Similar increases in the Bader charge and depletion in the d occupancies were observed for other high-pressure phases of TiO_2_ upon the application of the Hubbard correction (Table S2 in the Supplementary Materials). The Bader charges from the the r^2^SCAN+U calculations were identical to those obtained using the much larger value of U = 6 eV in the PBE+U calculations. In our previous study using the PBE+U method [41], we noted that the increased ionicity with the Hubbard correction is directly related to the relative stability of rutile and anatase. The enhanced ionic character of TiO_2_ due to the on-site U term increased the magnitude of the Madelung energy for both, as expected. However, the Madelung energy for rutile increased more rapidly, lowering the total energy further below that of anatase, therefore reversing the relative energetics between rutile and anatase.

A ramification of the small U used in the r^2^SCAN+U calculations was the smaller expansion of the bond distances for rutile and anatase due to the Hubbard correction. Several bond distances for rutile and anatase are compared between the r^2^SCAN+U and the PBE+U calculations using $R_{mt}$ (Ti) = 1.78 *a_B_* in Table 3. Both r^2^SCAN+U(2.5 eV) and the PBE+U(6 eV) calculation showed that the bond distance Ti-O(1) between Ti and apical O remained nearly identical to the values without the Hubbard U interaction term. However, the bond distance Ti-O(2) between Ti and equatorial O extended more pronouncedly with an increasing U. In our previous study [41], the increased Ti-O(2) bond length with a larger U value was attributed to the increased ionicity resulting from the additional charge transfer caused by the application of the U term. The elongated bond length especially contributed to the larger lattice constant *c* for rutile and larger lattice constants *a* and *b* for anatase. In the r^2^SCAN+U calculations, the lattice constants similarly increased with an increasing U value (Table S5 in the Supplementary Materials). However, because a smaller U value, e.g., 2.5 eV, was sufficient, the Ti-O(2) bond only increased by 0.005 to 0.006 Å for rutile and anatase, respectively, in the r^2^SCAN+U calculation. On the contrary, the PBE+U calculation with U = 6 eV increased the same bond length by about 0.013 to 0.014 Å.

Interestingly, the O-O bond distance along $\left[1\bar{1}0\right]$ in rutile remained nearly identical, increasing from 2.535 to 2.536 Å upon applying the Hubbard correction U = 2.5 eV in the r^2^SCAN+U calculation, just as it only increased by 0.003 Å in the PBE+U calculation. Little or no changes in the O-O and Ti-O(1) bond distances were consistent with the observation that the lattice constant *a* of rutile is relatively insensitive to the increase in U. However, the O-O bond distance of 2.560 Å obtained from the PBE+U calculation was significantly larger than the 2.536 Å obtained from the r^2^SCAN+U calculation, as well as the experimental value of 2.537 Å. For anatase, the O-O bond distance in the approximate equatorial plane increased modestly from 2.797 to 2.805 Å upon the application of U = 2.5 eV in the r^2^SCAN+U calculation. Using PBE+U with *U* = 6 eV, the O-O bond distance increased much more significantly from 2.822 to 2.839 Å. Consequently, the elongated O-O and Ti-O(2) bond lengths generated a markedly poor agreement in the lattice constants *a* and *b* of anatase with the corresponding experimental values, as the larger U value was used in the PBE+U calculation.

The overestimation of the bond lengths especially became worse when a larger projection space and a larger U value were employed together. For $R_{mt}$ (Ti) = 2.2 *a_B_*, the r^2^SCAN+U calculations for rutile and anatase still correctly predicted the higher stability of rutile over anatase with the small *U* = 2.5 eV. The Ti-O(1) and Ti-O(2) bond lengths for the corresponding rutile structure were slightly overestimated by 0.011 and 0.022 Å, respectively, in comparison with experimental values (Table 3). For anatase, the bond lengths were similarly greater by only 0.022 and 0.014 Å. Accordingly, the lattice constants increased rather moderately (Table S5 in the Supplementary Materials). On the other hand, for PBE+U, rutile became more stable than anatase with U = 7.7 eV. With the large U value, the PBE+U calculations overestimated the Ti-O(1) and Ti-O(2) bond lengths of the corresponding rutile by as much as 0.045 and 0.042 Å, respectively (Table 3). For anatase, the bond lengths were likewise substantially greater by 0.014 and 0.037 Å. The deviation of the structural parameters from the experimental values was exacerbated not only by the relatively large U value necessary for the PBE+U calculations but also by the larger $R_{mt}$(Ti) value used.

**Figure 8:**
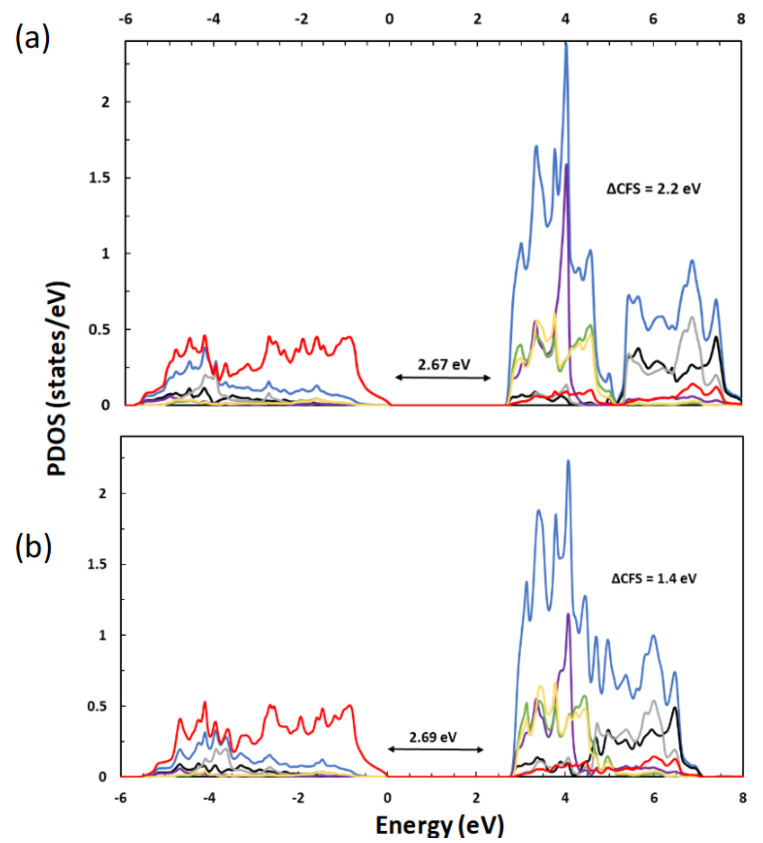
The projected density of states (PDOS) for rutile calculated using (**a**) r^2^SCAN+U (2.5 eV) and (**b**) the PBE+U (7.7 eV) functional with $R_{MT}$ (Ti) = 2.2 *a_B_*: Ti *d* (blue), $d_{z^{2}}$ (black), $d_{x^{2}-y^{2}}$ (gray), $d_{xy}$ (purple), $d_{yz}$ (yellow), $d_{zx}$ (green), and O *p* (red).

Finally, the smaller U value used in the r^2^SCAN+U calculations provided a better account of the crystal field splitting (CFS) between the $t_{2g}$ and *eg* bands in the electronic structure, compared to the larger U in the PBE+U calculation. Figure 8 exhibits the projected density of states, calculated using (a) the r^2^SCAN functional with *U* = 2.5 eV and (b) the PBE functional with *U* = 7.7 eV. The particular U values were chosen to have the correct stabilities of rutile and anatase shown in Figure 6b with the large projection space ($R_{mt}$ (Ti) = 2.2 *a_B_*). Both the r^2^SCAN+U and PBE+U methods produced almost identical band gaps of 2.67 and 2.69 eV, respectively, between the O 2*p*-dominated valence bands and mainly Ti 3*d*-derived conduction bands. However, the CFS between the $t_{2g}$ and *eg* bands showed a more pronounced contrast: 2.2 eV from the r^2^SCAN+U calculation vs. 1.4 eV from the PBE+U calculation. The difference results from the asymmetrical potential energy shifts between the $t_{2g}$ and *eg* bands upon the application of the Hubbard interaction [41]. The largely empty $t_{2g}$ bands shift by a much greater extent than the *e_g_* bands, which are substantially occupied as they participate in bonding with O. The uneven shift is proportional to the U value (Equation (6) in Section 3), so the PBE+U method, which requires a relatively large U value, produces a significantly larger deviation from the experimental value of 2.6 eV [76].

## 3. Methods

The calculations were carried out using the all-electron augmented plane wave (APW) and local orbital program, implemented in Wien2k [77,78]. In the APW scheme, the unit cell was divided into non-overlapping muffin-tin (MT) spheres and interstitial regions. Inside the MT spheres, which were centered at atoms, a linear combination of radial functions multiplied by spherical harmonics was used for basis functions. In the interstitial regions, a plane wave expansion was employed. The core states were fully localized within the MT sphere and distinguished from valence states with an energy separation of −6.0 Ry or larger. The value of the energy cutoff resulted in Ti 3s and 3p being semicore states and Ti 3d and 4s as well as O 2s and 2p being valence states. The core electrons were treated fully relativistically using the Dirac–Fock method. The valence electrons were treated with a scalar relativistic approach.

For the exchange–correlation effect, the PBE functional was used at the GGA level, serving as a reference. For the meta-GGA level, a regularized–restored strongly constrained appropriately normed (r^2^SCAN) functional [52] was utilized and compared to the PBE. r^2^SCAN makes use of the dimensionless variable $\bar{\alpha }$:

(2) $$\bar{\alpha }=\frac{\tau \left(r\right)-\tau_{W}\left(r\right)}{\tau_{UEG}\left(r\right)+\eta \tau_{W}\left(r\right)},$$

where $\tau \left(r\right)=\frac{1}{2}\sum_{i}{\left|\nabla \psi_{i}\right|}^{2}$ is the positive definite kinetic energy density with the Kohn–Sham orbitals $\psi_{i}\left(r\right)$. $\tau_{W}\left(r\right)={\left|\nabla n\right|}^{2}/8n$ is the von Weizsäcker kinetic energy density [42,44]. $\tau_{UEG}\left(r\right)=3{\left(3\pi^{2}\right)}^{2/3}n^{5/3}/10$ is the kinetic energy density of a uniform electron gas. $\bar{\alpha }$ is modified from $\alpha =\left(\tau -\tau_{W}\right)/\tau_{UEG}$ in SCAN by $\eta =10^{-3}$, which is a simple regularization parameter. The correct uniform and non-uniform scaling properties are obtained using the $\bar{\alpha }$-driven interpolation function as $\bar{\alpha }\to$ 1, 0, or *∞* for the metallic (uniform density), single covalent, or weak bond limits, respectively [42]. For the present study, the r^2^SCAN calculations were performed self-consistently. In the self-consistent implementation in Wien2k [79], the exchange–correlation potential was derived by taking the functional derivative with respect to the Kohn–Sham orbital, using the optimized effective potential method within the generalized Kohn–Sham (gKS) framework.

The muffin-tin radii ($R_{MT}$) for Ti and O were initially chosen to be 1.78 and 1.61 bohrs (*a_B_*), respectively. For the investigation of the structures compressed to high pressures, they were reduced by about 5%. The accuracy of the calculation was controlled by a dimensionless cutoff parameter, $R_{MT}K_{max}$, which determined the size of the basis from the product of the smallest $R_{MT}$ value and the largest K vector in the plane wave expansion. Because the energy difference between the TiO_2_ polymorphs can be small, the convergence of the calculated energy with respect to various parameters was carefully checked. The value of $R_{MT}K_{max}$ was increased up to 9 and the Brillouin Zone was sampled from 700 to 2000 points for convergence. For the PBE, a Fourier cutoff value of $G_{max}=16$ was sufficient, whereas for r^2^SCAN, a larger value, up to $G_{max}=28$, was tested to ensure the total energy convergence [79].

The electron correlation effect of *d* electrons was taken into account using the orbital-dependent potential formulated in the Hubbard on-site interaction term [80]. The interaction is based on a rotationally or unitary transformation-invariant formulation [81].

(3) $$\begin{array}{c} E_{Hub}\left[\{n_{mm^{\prime }}\}\right]=\frac{1}{2}\sum_{\{m\},\sigma }\{\langle m,m^{\prime \prime }|V_{ee}|m^{\prime },m^{\prime \prime \prime }\rangle n_{mm^{\prime }}^{\sigma }n_{m^{\prime \prime }m^{\prime \prime \prime }}^{-\sigma }\\ +(\langle m,m^{\prime \prime }|V_{ee}|m^{\prime },m^{\prime \prime \prime }\rangle -\langle m,m^{\prime \prime }|V_{ee}|m^{\prime \prime \prime },m^{\prime }\rangle )n_{mm^{\prime }}^{\sigma }n_{m^{\prime \prime }m^{\prime \prime \prime }}^{\sigma }\}, \end{array}$$

where $n_{mm^{\prime }}^{\sigma }$ is the density matrix for correlated electrons with the magnetic quantum number of the orbital angular momentum *l* and spin index σ, and $V_{ee}$ are the screened Coulomb interactions. Because the standard density functional approximations already include the part of the interaction energy which the Hubbard model aims to better describe, it must be subtracted via the double-counting term $E_{dc}$. The most widely employed method of eliminating double counting for a strongly correlated insulating system was chosen [80]:

(4) $$E_{dc}\left[\{n_{mm^{\prime }}\}\right]=\frac{U}{2}n\left(n-1\right)-\frac{J}{2}\left[n^{↑}\left(n^{↑}-1\right)-n^{↓}\left(n^{↓}-1\right)\right],$$

where $n^{\sigma }=Tr\left(n_{mm^{\prime }}^{\sigma }\right)$ and $n=n^{↑}+n^{↓}$ are the total occupation numbers of localized orbitals.

The interaction energy can be further simplified by keeping only the lowest order Slater integral *F*^0^ [67,82] when evaluating the interaction matrix elements $\langle m,m^{\prime \prime }|V_{ee}|m^{\prime },m^{\prime \prime \prime }\rangle$. Under this isotropic scheme of the DFT+U, the Hubbard correction with the double-counting term included is written as

(5) $$E_{Hub}=\sum_{\sigma }\frac{U_{eff}}{2}Tr\left[n^{\sigma }\left(1-n^{\sigma }\right)\right]$$

with $U_{eff}$ regarded as an effective parameter including the exchange *J*, such that $U_{eff}=U-J$. Consequently, through differentiation with respect to each orbital occupancy, one obtains the additional orbital-dependent potential energy:

(6) $$V_{m}^{\sigma }=U_{eff}\left(\frac{1}{2}-n_{m}^{\sigma }\right)$$

In this study, the orbital-dependent correction obtained using Equation (6) was applied to the localized orbitals described by the basis function within the augmentation sphere defined by $R_{MT}$. Throughout the paper, $U_{eff}$ is referred to simply as U.

For PBE and PBE+U calculations, the lattice parameters and the ionic positions were fully relaxed. The internal coordinates were optimized by minimizing the forces (1 mRy/au) acting on the atoms. For r^2^SCAN, the calculation of forces was not possible due to the fact that the core and valence electrons were treated using inconsistent potentials as implemented in Wien2k [79]. For the core electrons, a GGA exchange–correlation potential was used to obtain the Hamiltonian matrix elements. However, the valence electrons were treated in a fully self-consistent manner using the meta-GGA potential in the gKS scheme. Hence, only the lattice parameters were optimized for r^2^SCAN and r^2^SCAN+U calculations, using the internal coordinates derived from PBE and PBE+U calculations.

## 4. Conclusions

In summary, we have investigated various phases of TiO_2_ across different pressure ranges using the r^2^SCAN functional and performed the Hubbard-corrected r^2^SCAN+U calculations to study the relative stability between rutile and anatase in particular. The equilibrium lattice parameters calculated by the r^2^SCAN functional were generally in excellent agreement with the experimental values for the TiO_2_ phases. The total energy calculations produced an order of stability of anatase < rutile < columbite < baddeleyite < orthorhombic *I* < cotunnite, as in the calculations using the PBE functional. However, the inclusion of the Hubbard correction term with a much smaller value of U, compared to the PBE+U calculations, yielded the correct ordering of rutile and anatase. Although the r^2^SCAN+U method also showed a dependence on the size of the local projection space used in the Hubbard correction, the dependence was much less sensitive compared to the PBE+U method. The relatively weak sensitivity to the projection space size is attributed to the improvement in the r^2^SCAN functional, permitting a smaller U value to be used. The better description of the structural parameters, electronic structures, and charge distributions afforded by a relatively modest value of U suggests that the r^2^SCAN functional can be a promising choice in the Hubbard-corrected DFT approach.

## Figures and Tables

**Table 1:** The lattice parameters of six TiO_2_ phases, calculated at *T* = 0 K and *p* = 0 GPa unless stated otherwise. The lattice constants are in Å, and the angle β of the monoclinic phase is in ^∘^.

		Lattice Constants			
**Phase**	***a***	***b* (*β*)**	***c***	$B_{0}$ ** (GPa)**	**Reference**
rutile	4.600		2.956	244	this work (r^2^SCAN)
	4.646		2.966	214	this work (PBE)
	4.591		2.957		other (SCAN) [53]
	4.655		2.970	210	other (PBE) [32]
	4.593 ^*a*^		2.959 ^*a*^	235 ^*b*^, 211 ^*c*^, 210 ^*d*^	expt.
anatase	3.779		9.601	212	this work (r^2^SCAN)
	3.805		9.716	188	this work (PBE)
	3.777		9.587		other (SCAN) [53]
	3.806		9.737	170	other (PBE) [32]
	3.785 ^*a*^		9.514 ^*a*^	179 ^*c*^, 190 ^*c*^	expt.
columbite	4.549	5.521	4.897	254	this work (r^2^SCAN)
	4.580	5.576	4.927	206	this work (PBE)
	4.537	5.504	4.905		other (SCAN) [53]
	4.588	5.590	4.933	214	other (PBE) [32]
	4.541 ^*c*^	5.493 ^*c*^	4.906 ^*c*^	258 ^*c*,*d*^, 253 ^*b*^, 206 ^*e*^	expt.
baddeleyite	4.836	4.857	5.059	273	this work (r^2^SCAN)
		(100.3)			
	4.861	4.906	5.111	171	this work (PBE)
		(100.3)			
	4.800	4.867	5.026		other (SCAN) [53]
		(99.82)			
	4.866	4.920	5.108	149	other (PBE) [32]
		(99.9)			
	4.662 ^*c*^	4.969 ^*c*^	4.911 ^*c*^	304 ^*f*^, 298 ^*b*^, 290 ^*d*^, 175 ^*e*^	expt.
		(99.4 ^*c*^)			
OI	9.358	4.950	4.801	280	this work (r^2^SCAN)
	9.426	4.986	4.842	221	this work (PBE)
	9.428	4.985	4.837	247	other (PBE) [32]
	9.046 ^*g*,*h*^	4.834 ^*g*,*h*^	4.621 ^*g*,*h*^	318 ^*g*^, 314 ^*b*^, 222 ^*e*^	expt.
	9.037 ^*h*^	4.832 ^*h*^	4.629 ^*h*^	283	this work (r^2^SCAN)
	9.071 ^*h*^	4.864 ^*h*^	4.654 ^*h*^	229	this work (PBE)
	9.138 ^*h*^	4.853 ^*h*^	4.671 ^*h*^	252	other (B3LYP) [33]
cotunnite	5.169	3.167	6.290	297	this work (r^2^SCAN)
	5.217	3.185	6.329	184	this work (PBE)
	5.231	3.151	6.261	270	other (PBE) [32]
	5.240	3.163	6.297		other (PBE) [54]
	5.18	3.191	6.328	213	other (PW91) [29]
	5.125 ^*i*^	2.914 ^*i*^	5.931 ^*i*^	255	other (B3LYP) [33]
	5.163 ^*f*,*j*^	2.989 ^*f*,*j*^	5.966 ^*f*,*j*^	431 ^*f*^, 312 ^*b*^, 294 ^*e*^	expt.

^*a*^ Ref. [14]; ^*b*^ Ref. [28]; ^*c*^ Ref. [17]; ^*d*^ Ref. [57]; ^*e*^ Ref. [58]; ^*f*^ Ref. [19]; ^*g*^ Ref. [18]; ^*h*^ at 28 GPa; ^*i*^ at 60 GPa; ^*j*^ at 61 GPa.

**Table 2:** Bader charges and bond critical points calculated using the PBE and r^2^SCAN XC functional with and without U corrections ($R_{MT}$ (Ti) = 1.78 *a_B_*). For each BCP along Ti-O(1) (apical O) and Ti-O(2) (equatorial O), as defined in [41], the two distances from Ti and O are listed.

**Rutile**		**Bader Charge (*e*)**		**BCP (3, −1) (in Å)**	
**XC Functional**	**U (** eV **)**	**Ti**	**O**	**Ti; O(1)**	**Ti; O(2)**
PBE	0	+2.28	−1.14	1.002; 1.004	0.982; 0.977
PBE+U	6	+2.46	−1.23	1.003; 1.015	0.983; 0.985
r^2^SCAN	0	+2.38	−1.19	0.995; 0.996	0.976; 0.968
r^2^SCAN+U	2.5	+2.46	−1.23	0.993; 1.000	0.976; 0.974
**Anatase**		**Bader Charge (e)**		**BCP** (3,−1) **(in Å)**	
**XC Functional**	**U (*eV*)**	**Ti**	**O**	**Ti; O(1)**	**Ti; O(2)**
PBE	0	+2.26	−1.13	0.997; 0.998	0.975; 0.974
PBE+U	6	+2.44	−1.22	1.001; 1.003	0.971; 0.978
r^2^SCAN	0	+2.37	−1.19	0.993; 0.994	0.965; 0.969
r^2^SCAN+U	2.5	+2.44	−1.22	0.991; 0.997	0.969; 0.971

**Table 3:** The two inequivalent Ti-O bond lengths and O-O bond distances for rutile and anatase are listed (in Å) with and without the Hubbard U correction, calculated using the r^2^SCAN and PBE functionals. For the calculations with R_*MT*_ (Ti) = 1.78 *a_B_*, U values of 2.5 and 6 eV were used, respectively, resulting in approximately the same Bader charges. For R_*MT*_ (Ti) = 2.2 *a_B_*, U values of 2.5 and 7.7 eV resulted in the correct ordering of the relative stability of rutile and anatase.

	Rutile			Anatase		
**Ti R_*MT*_: 1.78 *a^B^***	**Ti-O(1)**	**Ti-O(2)**	**O-O ^a^**	**Ti-O(1)**	**Ti-O(2)**	**O-O ^b^**
r^2^SCAN	1.986	1.947	2.535	1.987	1.934	2.797
PBE	2.005	1.959	2.560	2.004	1.949	2.822
r^2^SCAN+U (2.5 eV)	1.986	1.952	2.536	1.988	1.940	2.805
PBE+U (6 eV)	2.007	1.973	2.563	2.005	1.962	2.839
Ti R_*MT*_: 2.2 *a_B_*						
r^2^SCAN	1.982	1.944	2.531	1.987	1.939	2.803
PBE	2.006	1.956	2.556	2.007	1.949	2.822
r^2^SCAN+U (2.5 eV)	1.991	1.971	2.541	1.991	1.950	2.820
PBE+U (7.7 eV)	2.025	1.991	2.572	2.022	1.983	2.869
Expt [14]	1.980	1.949	2.537	1.969	1.936	2.799

^a^ [110]; ^b^ in equatorial plane.

## Data Availability

The original contributions presented in this study are included in the article/Supplementary Material. Further inquiries can be directed to the corresponding author.

## Supplementary Materials

The following supporting information can be downloaded at https://www.mdpi.com/article/10.3390/molecules30030560/s1.
