# Supplementary material for: The Stability of TiO2 Phases Studied Using r2SCAN in the Hubbard-Corrected Density Functional Theory
Source: Molecules. 2025 Jan 26;30(3):560. doi: 10.3390/molecules30030560 (PMC11820048; doi:10.3390/molecules30030560)
Supplement: Supplementary file 1 [file molecules-30-00560-s001.zip › molecules-3410395-supplementary.pdf]

Supplementary Materials to "Stability of TiO_2 studied using r^2SCAN in the Hubbard-corrected DFT"

Jared Pohlmann, Manjula Raman, Lily Bonds, Kenneth Park
Department of Physics, Baylor University, Waco, Texas, USA

Table S1. The Wyckoff positions are listed for the six optimized TiO_2 structures at T=0 K and p = 0 GPa unless stated otherwise.

Phase	atomic positions<br>Ti	O	reference
rutile	2a (0, 0, 0)	4f (0.305, 0.305, 0)	this work (r^2SCAN)
	2a (0, 0, 0)	4f (0.305, 0.305, 0)	this work (PBE)
	2a (0, 0, 0)	4f (0.305, 0.305, 0)	other (PBE)[1]
	2a (0, 0, 0)	4f (0.306, 0.306, 0)	Expt. [2]
anatase	4b (0, 0.250, 0.375)	8e (0, 0.250, 0.168)	this work (r^2SCAN)
	4b (0, 0.250, 0.375)	8e (0, 0.250, 0.168)	this work (PBE)
	4b (0, 0, 0)	8e (0, 0, 0.206)	other (PBE)[1]
	4a(0,0,0)	8e (0, 0, 0.207)	Expt. [2]
columbite	4c (0, 0.323, 0.250)	8d (0.271, 0.119, 0.081)	this work (r^2SCAN)
	4c (0, 0.323, 0.250)	8d (0.271, 0.119, 0.081)	this work (PBE)
	4c (0, 0.177, 0.250)	8d (0.271, 0.381, 0.419)	other (PBE) [1]
	4c (0, 0.173, 0.25)	8d (0.286, 0.376, 0.412)	Expt. [3]
baddeleyite	4e (0.274, 0.059, 0.219)	4e (0.057, 0.315, 0.360)<br>4e (0.453, 0.759, 0.450)	this work (r^2SCAN)
	4e (0.274, 0.059, 0.219)	4e (0.057, 0.315, 0.360)<br>4e (0.453, 0.759, 0.450)	this work (PBE)
	4e (0.276, 0.058, 0.217)	4e (0.062, 0.321, 0.355)<br>4e (0.449, 0.759, 0.459)	other (PBE) [1]
	4e (0.279, 0.047, 0.209)	4e (0.078, 0.354, 0.329)<br>4e (0.444, 0.761, 0.485)	other (LDA) [4]
OI	8c (0.886, 0.042, 0.256)	8c (0.795, 0.389, 0.158)<br>8c (0.968, 0.736, 0.493)	this work (r^2SCAN)
	8c (0.886, 0.042, 0.256)	8c (0.795, 0.389, 0.158)<br>8c (0.968, 0.736, 0.493)	this work (PBE)
	8c (0.885, 0.042, 0.249)	8c (0.789, 0.375, 0.138)<br>8c (0.975, 0.737, 0.496)	other (PBE) [1]
	8c (0.885, 0.049, 0.256)^a	8c (0.805, 0.401, 0.135)^a<br>8c (0.945, 0.690, 0.464)^a	Expt. [5]
	8c (0.886, 0.042, 0.255)^a	8c (0.794, 0.385, 0.154)^a<br>8c (0.970, 0.736, 0.494)^a	this work (r^2SCAN)
	8c (0.886, 0.042, 0.255)^a	8c (0.794, 0.385, 0.154)^a<br>8c (0.970, 0.736, 0.494)^a	this work (PBE)
cotunnite	4c (0.254, 0.250, 0.102)	4c (0.364, 0.250, 0.416)<br>4c (0.011, 0.750, 0.349)	this work (r^2SCAN)
	4c (0.254, 0.250, 0.102)	4c (0.364, 0.250, 0.416)<br>4c (0.011, 0.750, 0.349)	this work (PBE)
	4c (0.251, 0.25, 0.107)	4c (0.363, 0.25, 0.419)<br>4c (0.016, 0.75, 0.346)	other (PBE) [1]
	4c (0.264, 0.25, 0.110)^b	4c (0.346, 0.25, 0.422)^b<br>4c (0.012, 0.75, 0.325)^b	Expt. [6]

^{a} at 28 GPa; ^{b} at 61 GPa

Figure S1. The total energy of OI is plotted as a function of volume using (a) & (b) the r^{2}SCAN and (c) & (d) the PBE functional. To simulate the structure of OI phase at 28 GPa, the corresponding volume is first obtained from the BM fit. Then, the lattice constants *a*, *b*, and *c* are relaxed with the fixed volume ((a) & (c)). Finally, the ratios of *c/a* and *b/a* are further optimized ((b) & (d)).

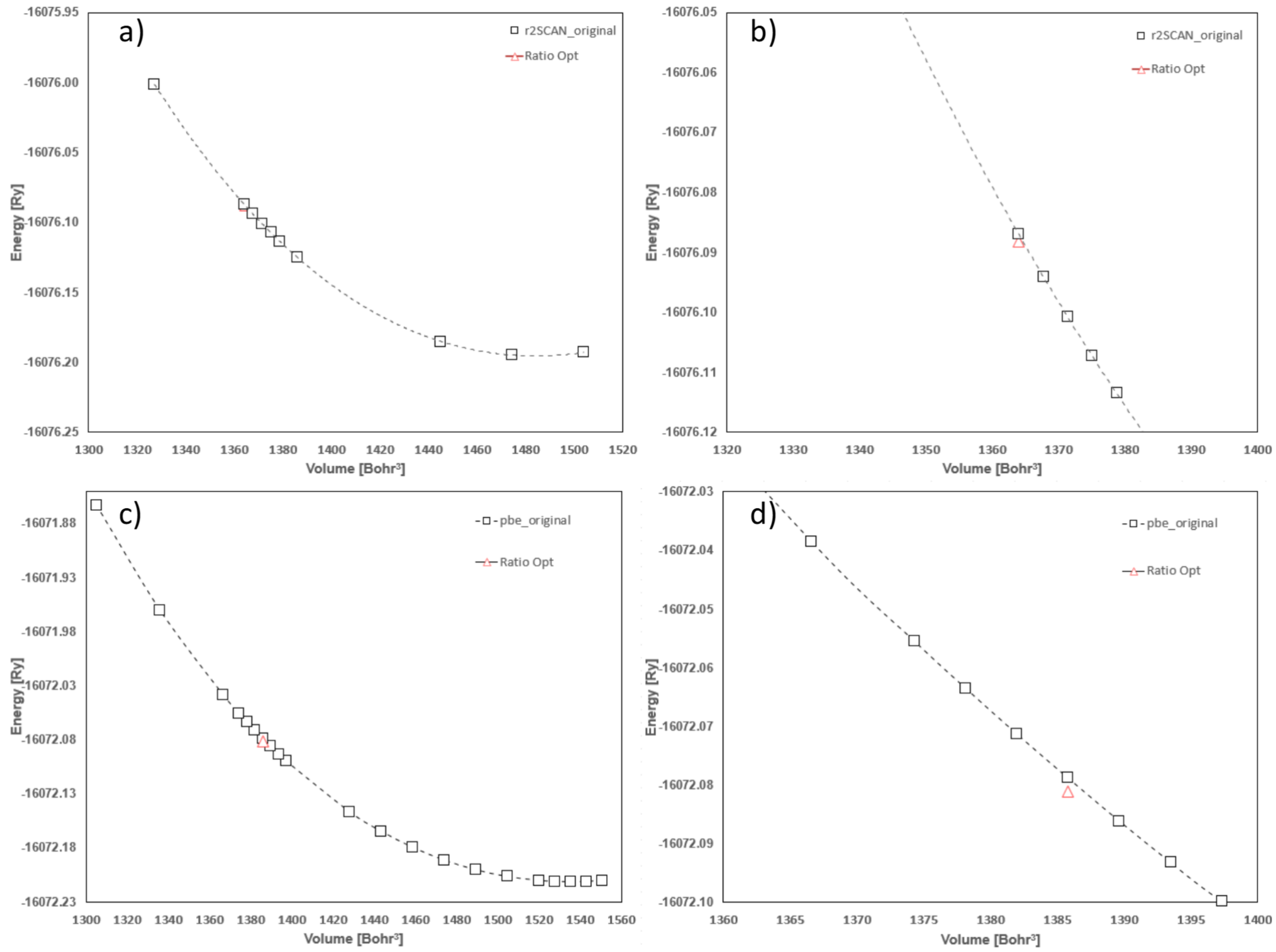

Figure S2. Total energy of TiO_2 as a function of volume for various phases calculated using the PBE functional. Each symbol represents a full structural optimization: rutile (dark blue diamond), anatase (red square), columbite (green triangle), baddeleyite (purple cross), OI (blue cross), and cotunnite (orange circle). The solid line is fitted with the Birch-Murnaghan equation of state.

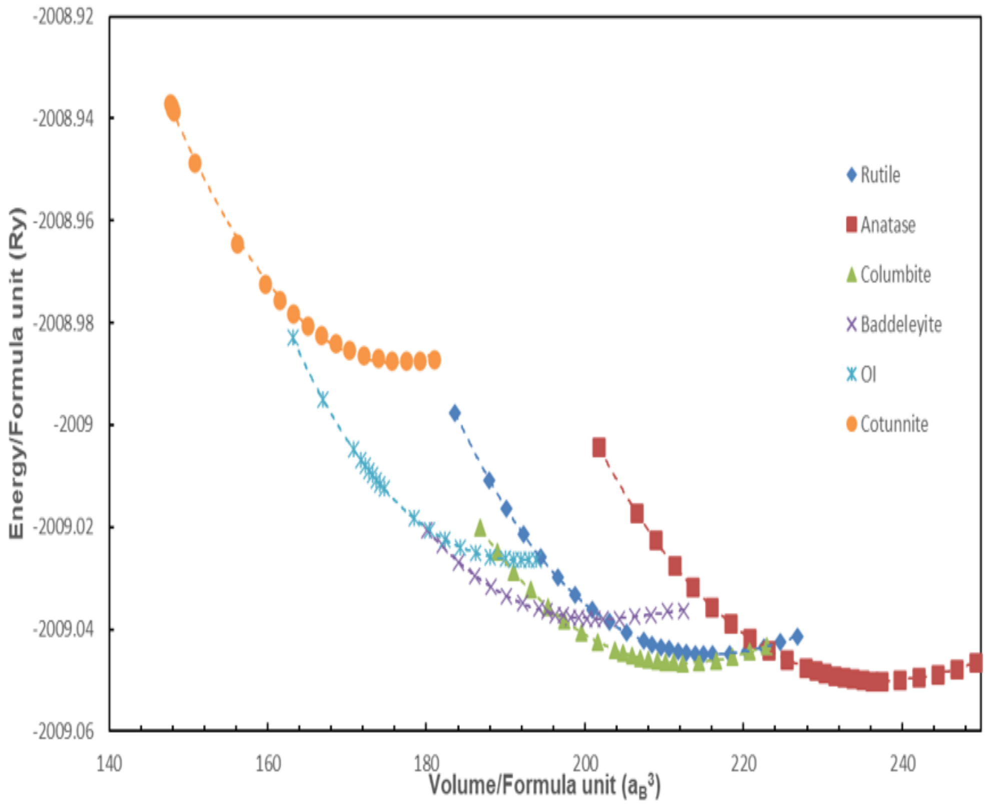

Table S2. The total occupancies of *d* orbitals (per spin) n_d and Bader charges of Q(Ti) for Ti and Q(Oj) (j=1,2 for inequivalent oxygen if applicable) calculated using the PBE, r^2SCAN and r^2SCAN+U(2.5eV) functionals for rutile, anatase, columbite, baddeleyite, OI, and cotunnite in their equilibrium structures. R_{MT}(Ti) = 1.78 a_B is used.

XC	Occupancy/ charges	Rutile	Anatase	Columbite	Baddeleyite	OI	Cotunnite
PBE	n_d	0.54	0.55	0.55	0.56	0.55	0.56
	Q(Ti)	2.28	2.26	2.27	2.25	2.26	2.22
	Q(O1)	-1.14	-1.13	-1.14	-1.09	-1.11	-1.11
	Q(O2)				-1.16	-1.14	-1.12
r^2SCAN	n_d	0.52	0.53	0.53	0.54	0.53	0.54
	Q(Ti)	2.38	2.37	2.36	2.34	2.35	2.32
	Q(O1)	-1.19	-1.19	-1.18	-1.14	-1.16	-1.15
	Q(O2)				-1.20	-1.18	-1.16
r^2SCAN + U(2.5eV)	n_d	0.49	0.50	0.50	0.51	0.50	0.51
	Q(Ti)	2.46	2.44	2.44	2.41	2.43	2.39
	Q(O1)	-1.23	-1.22	-1.22	-1.17	-1.20	-1.19
	Q(O2)				-1.24	-1.22	-1.20

Figure S3. The projected density of states for rutile with the r^{2}SCAN (top) and the PBE (bottom) functionals: Ti total d (blue), d_{z2} (black), d_{x2-y2} (gray), d_{xy} (purple), d_{yz} (yellow), d_{zx} (green), O p (red). The fermi level is arbitrary set to the top of the valance band maximum. Also, the band gap energy and the crystal field splitting between t_{2g} and e_g are indicated.

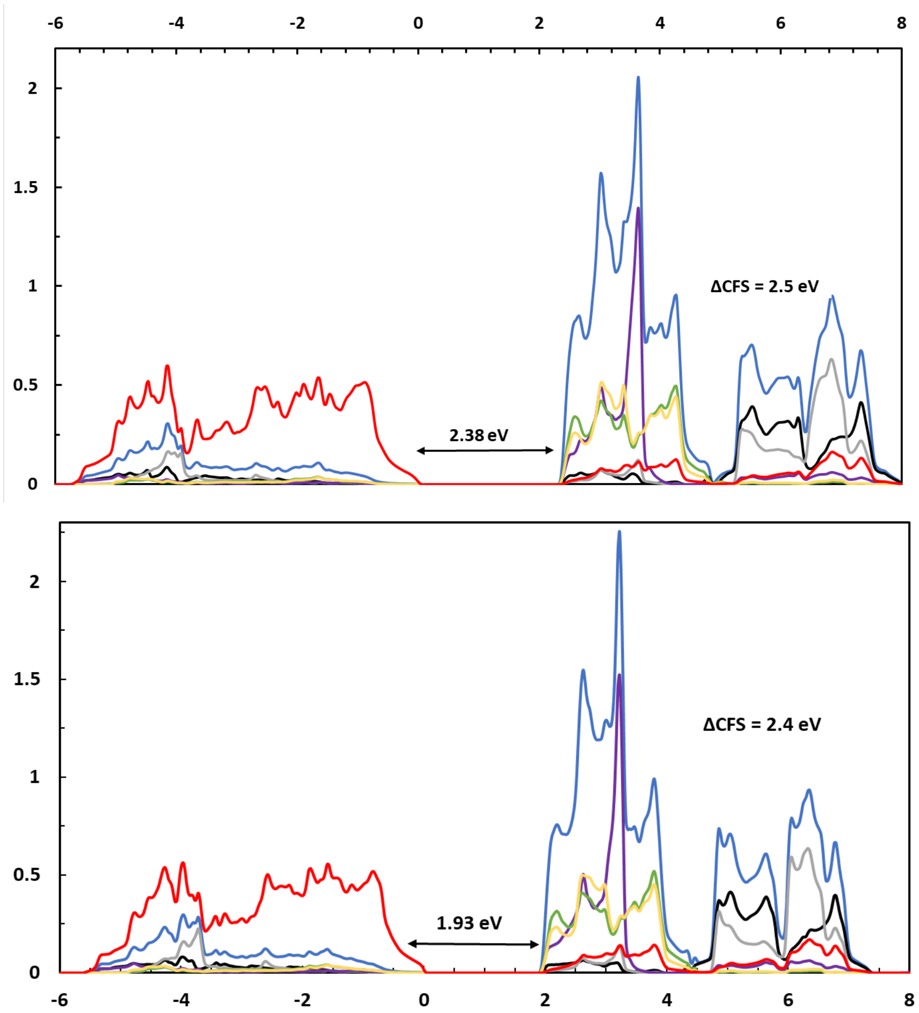

Table S3. The occupancies of *d* orbitals (per spin) of rutile, calculated by integrating PDOS, are compared between the r^{2}SCAN and the PBE functionals as well as with or without U. R_{MT}(Ti) = 1.78 a_B is used.

**Rutile**	d	dz2	d(x2-y2)	dxy	dxz	dyz
r2SCAN	0.494	0.138	0.148	0.065	0.072	0.072
r2SCAN+U(2.5eV)	0.442	0.126	0.136	0.056	0.062	0.062
PBE	0.526	0.141	0.155	0.071	0.08	0.079
PBE+U(6eV)	0.446	0.128	0.138	0.054	0.063	0.062

Figure S4. The projected density of states for anatase with the r^2SCAN (top) and the PBE (bottom) functionals: Ti total d (blue), d_{z2} (black), d_{x2-y2} (gray), d_{xy} (purple), d_{yz} (yellow), d_{zx} (green), O p (red). The fermi level is arbitrary set to the top of the valance band maximum. Also, the band gap energy and the crystal field splitting between t_{2g} and e_g are indicated.

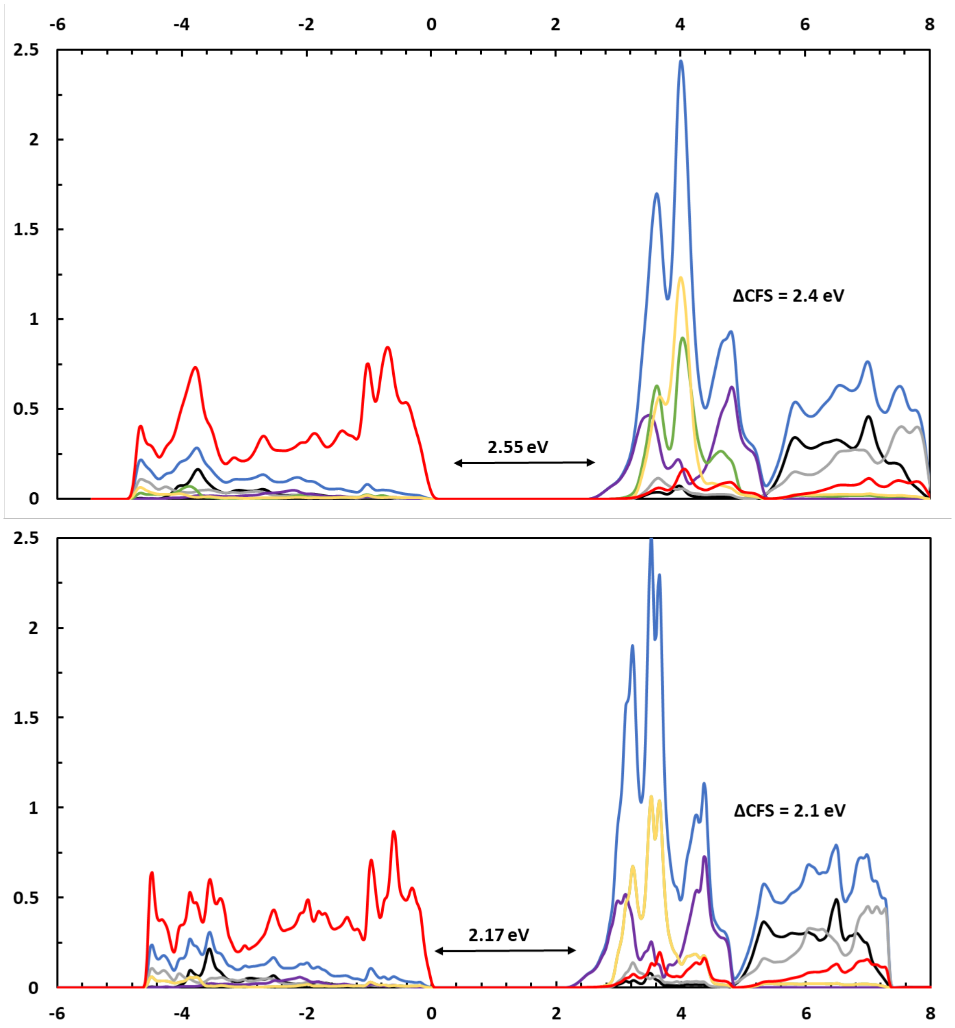

Table S4. The occupancies of *d* orbitals (per spin) of anatase, calculated by integrating PDOS, are compared between the r^2SCAN and the PBE functionals as well as with or without U. R_{MT}(Ti) = 1.78 a_B is used.

**Anatase**						
	d	dz2	d(x2-y2)	dxy	dxz	dyz
r2SCAN	0.506	0.145	0.143	0.076	0.076	0.066
r2SCAN+U(2.5eV)	0.474	0.139	0.137	0.07	0.068	0.06
PBE	0.523	0.148	0.143	0.083	0.075	0.075
PBE+U(6eV)	0.478	0.141	0.132	0.067	0.069	0.069

Table S5. Lattice parameters for rutile and anatase for varying values of Hubbard U (eV) and Ti R_{mt} using the r^2SCAN functional. The percent change in lattice parameters references the difference between U=0 eV and other U values.

**Rutile**					
	U Value	a	c	% a	% c
Ti 1.78	0	4.600	2.956	0.00	0.00
	2.5	4.602	2.970	0.04	0.47
	6	4.608	2.989	0.17	1.12
	10	4.615	3.008	0.33	1.76
Ti 1.99	0	4.602	2.957	0.00	0.00
	2.5	4.608	2.976	0.13	0.64
	6	4.616	3.000	0.30	1.45
	10	4.629	3.025	0.59	2.30
Ti 2.2	0	4.593	2.952	0.00	0.00
	2.5	4.584	2.988	-0.20	1.22
	6	4.632	3.027	0.85	2.54
	10	4.653	3.055	1.31	3.49
**Anatase**					
	U Value	a	c	% a	% c
Ti 1.78	0	3.779	9.601	0.00	0.00
	2.5	3.792	9.603	0.34	0.02
	6	3.810	9.621	0.82	0.21
	10	3.826	9.644	1.24	0.45
Ti 1.99	0	3.781	9.597	0.00	0.00
	2.5	3.799	9.607	0.48	0.10
	6	3.823	9.633	1.11	0.38
	10	3.846	9.671	1.72	0.77
Ti 2.2	0	3.787	9.597	0.00	0.00
	2.5	3.812	9.618	0.66	0.22
	6	3.845	9.664	1.53	0.70
	10	3.880	9.724	2.46	1.32

References

[1] Zhao Fu, Yongcheng Liang, Shiming Wang, and Zheng Zhong. Structural phase transition and mechanical properties of tio2 under high pressure. Physica Status Solidi (b), 250(10):2206–2214, 2013.

[2] Don T Cromer and K Herrington. The structures of anatase and rutile. Journal of the American Chemical Society, 77(18):4708–4709, 1955.

[3] PY Simons and FJAC Dachille. The structure of tio2ii, a high-pressure phase of tio2. Acta Crystallographica, 23(2):334–336, 1967.

[4] Joseph Muscat, Varghese Swamy, and Nicholas M Harrison. First-principles calculations of the phase stability of tio 2. Physical Review B, 65(22):224112, 2002.

[5] Natalia A Dubrovinskaia, Leonid S Dubrovinsky, Rajeev Ahuja, Vitaly B Prokopenko, V Dmitriev, H-P Weber, JM Osorio-Guillen, and B¨orje Johansson. Experimental and theoretical identification of a new high-pressure tio 2 polymorph. Physical Review Letters, 87(27):275501, 2001.

[6] Leonid S Dubrovinsky, Natalia A Dubrovinskaia, Varghese Swamy, Joseph Muscat, Nicholes M Harrison, Rajeev Ahuja, Borja Holm, and Brje Johansson. The hardest known oxide. Nature, 410(6829):653–654, 2001.
